# How does mTOR sense glucose starvation? AMPK is the usual suspect

**DOI:** 10.1038/s41420-020-0260-9

**Published:** 2020-04-22

**Authors:** Gabriel Leprivier, Barak Rotblat

**Affiliations:** 1 Institute for Neuropathology, Medical Faculty, Heinrich Heine University, Moorenstr. 5, 40225 Düsseldorf, Germany; 2 Department of Life Sciences, Ben-Gurion University of the Negev, Beer-Sheva, 84105 Israel

**Keywords:** Cell biology, Cell signalling

## Abstract

Glucose is a major requirement for biological life. Its concentration is constantly sensed at the cellular level, allowing for adequate responses to any changes of glucose availability. Such responses are mediated by key sensors and signaling pathway components that adapt cellular metabolism to glucose levels. One of the major hubs of these responses is mechanistic target of rapamycin (mTOR) kinase, which forms the mTORC1 and mTORC2 protein complexes. Under physiological glucose concentrations, mTORC1 is activated and stimulates a number of proteins and enzymes involved in anabolic processes, while restricting the autophagic process. Conversely, when glucose levels are low, mTORC1 is inhibited, in turn leading to the repression of numerous anabolic processes, sparing ATP and antioxidants. Understanding how mTORC1 activity is regulated by glucose is not only important to better delineate the biological function of mTOR, but also to highlight potential therapeutic strategies for treating diseases characterized by deregulated glucose availability, as is the case of cancer. In this perspective, we depict the different sensors and upstream proteins responsible of controlling mTORC1 activity in response to changes in glucose concentration. This includes the major energy sensor AMP-activated protein kinase (AMPK), as well as other independent players. The impact of such modes of regulation of mTORC1 on cellular processes is also discussed.

## Facts

- mTORC1 is inhibited by AMPK-dependent and -independent mechanisms upon glucose depletion.
- mTORC1 recruitment to the lysosomal membrane is critical for mTORC1 activation in response to glucose.
- mTORC1 accommodates the activity of key anabolic processes to glucose availability.

## Open questions

- How do the known glucose sensors actually sense glucose and what are the other glucose sensors governing mTORC1 activity?
- Is the mTOR response to glucose availability quantitative or qualitative?
- How can we take advantage of the upstream regulation of mTORC1 by glucose to design novel anticancer strategies?

Glucose fuels organismal life. Organisms have evolved sophisticated biological mechanisms to sense and respond to changes in glucose availability. At the cellular level, there are key molecules that sense glucose levels and control the activity of specific signaling pathways that adapt cellular metabolism to the amount of available glucose.

One of the major hubs of glucose-sensing pathways is the highly conserved mechanistic target of rapamycin (mTOR) kinase, which is found in one or both of the protein complexes mTORC1/mTORC2 (ref. ^1^). During periods of glucose availability, mTORC1 is activated and phosphorylates a number of downstream targets to stimulate anabolic processes, including protein, nucleotide, and lipid syntheses, while blocking the catabolic process of autophagy^2^. This promotes mTORC1-driven cell growth and proliferation^3,4^. During times of glucose scarcity, mTORC1 is inhibited, leading to the blocking of the above-mentioned anabolic processes in conjunction with an induction of autophagy, resulting in the restriction of cell growth and proliferation^2^. This response is critical to preserve energy—protein synthesis being the most ATP consuming process in the cell^5^—as well as antioxidants, and therefore to preserve cell viability under such stress condition^6^. Indeed, failure to inactivate mTORC1 under glucose-deprived conditions leads to ATP depletion, in part due to abnormal protein synthesis activity, and cell death, indicating that mTORC1 inhibition is absolutely required to support cell survival during glucose shortage^7–9^.

The regulation of mTORC1 by glucose has pathological implications, as mTORC1 has been found to be deregulated in diseases characterized by abnormal glucose metabolism^10^. This is the case in cancer, whose microenvironment is characterized by poor glucose supply due to defective and inefficient tumor vasculature^11^. Since mTORC1 has been reported to be consistently overactive in various cancers^10^, and based on its pro-anabolic properties, it has been proposed as a therapeutic target for these diseases. While a number of mTORC1 inhibitors have been tested in a wide range of cancer types, their usage in clinics is currently rather limited^12^, in particular due to emergence of resistance^13^. Additionally, this is likely explained by the observation that mTORC1 inhibition mediates tumor cells protection against conditions of glucose deprivation^7,8^, commonly encountered within the tumor microenvironment. This was well illustrated by Palm et al., who demonstrated that in a mouse model of pancreatic cancer, the mTORC1 inhibitor rapamycin rather promotes proliferation of tumor cells located in poorly vascularized areas of the tumor^14^. Therefore, taking advantage of the current understanding of the regulation of mTORC1 by glucose, an attractive anticancer strategy would be to interfere with the repression of mTORC1 activity under glucose deprivation to prevent metabolic adaptation mediated by mTORC1 inhibition.

An important question that remains is how mTORC1 activity is controlled by glucose levels and which sensors are involved. While this has been well characterized in the case of amino acids, there is currently no clear overall picture for mTORC1 control in response to glucose.

Here, we depict the currently known upstream components and regulators of mTORC1 activity in response to glucose, offering possible suspects for the role of the glucose deprivation sensors that could represent potential therapeutic targets.

## AMPK

**Fig. 1:**
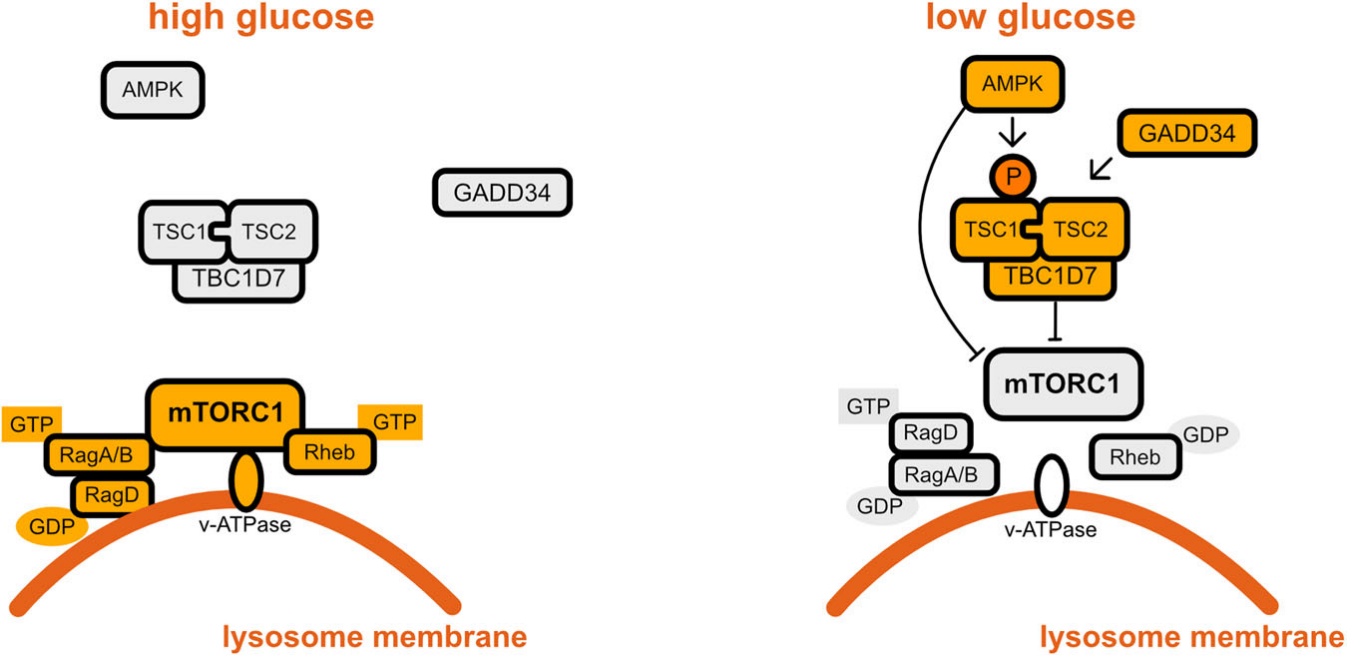
AMPK, Rag GTPases, GADD34, and TBC1D7 regulation of mTORC1 in response to glucose levels. Orange corresponds to active molecules; gray corresponds to inactive molecules.

One of the best characterized upstream regulators of mTORC1 activity in response to glucose is the energy sensor AMP-activated protein kinase (AMPK). Under glucose shortage, which induces energy depletion, AMPK directly senses increases in AMP:ATP and ADP:ATP ratios, leading to its activation^15–17^. The current model is that AMPK inhibits mTORC1 in response to glucose starvation through the phosphorylation and activation of the mTOR negative regulator tuberous sclerosis complex 2 (TSC2)^18^, on one hand, and through the phosphorylation and inhibition of the mTORC1 component regulatory-associated protein of mTOR (Raptor)^19^, on the other hand (Fig. 1). Such regulation was demonstrated to inhibit protein synthesis and cell cycle progression, controlling cell size and preventing apoptosis, downstream of mTORC1 inhibition, at times of energy crisis^7,18,19^. These findings provide further support to the model whereby mTORC1 senses glucose deprivation through AMPK. Nevertheless, in AMPK knockout cells, mTORC1 is still inactivated upon glucose starvation, indicating that AMPK is not essential for glucose sensing by mTORC1 (refs. ^20,21^) and therefore that other sensors of glucose levels control mTORC1.

## Rag GTPases and lysosomal v-ATPase

mTORC1 is regulated by a complex of the Ras-related GTPases (RagA–D), functioning as heterodimers of RagA or B interacting with RagC or D, with an important function in the response to amino acids^10^. Upon GTP loading of RagA and B, and GDP loading of RagC and D, mTORC1 is recruited to the surface of the lysosome where it is activated by Rheb^22^. This latter protein is a major regulator of mTORC1 activity that is directly under the repressive control of TSC1/2 (ref. ^23^). Some evidence suggests that Rag GTPases may also contribute to mTORC1 regulation by glucose. Indeed, overexpression of constitutively active GTP-bound RagA was found to increase cell sensitivity to glucose starvation, due to constitutive recruitment of mTORC1 to lysosomal membranes, and consequent activation by the lysosomal vacuolar-type H^+^-ATPase (v-ATPase^22^; Fig. 1). Furthermore, upon glucose starvation, v-ATPase recruits and activates AMPK, suggesting a mechanism linking the two kinases^24^. Nevertheless, how v-ATPase senses glucose starvation is not clear.

## ULK1 and the tRNA synthetase LARS1

**Fig. 2:**
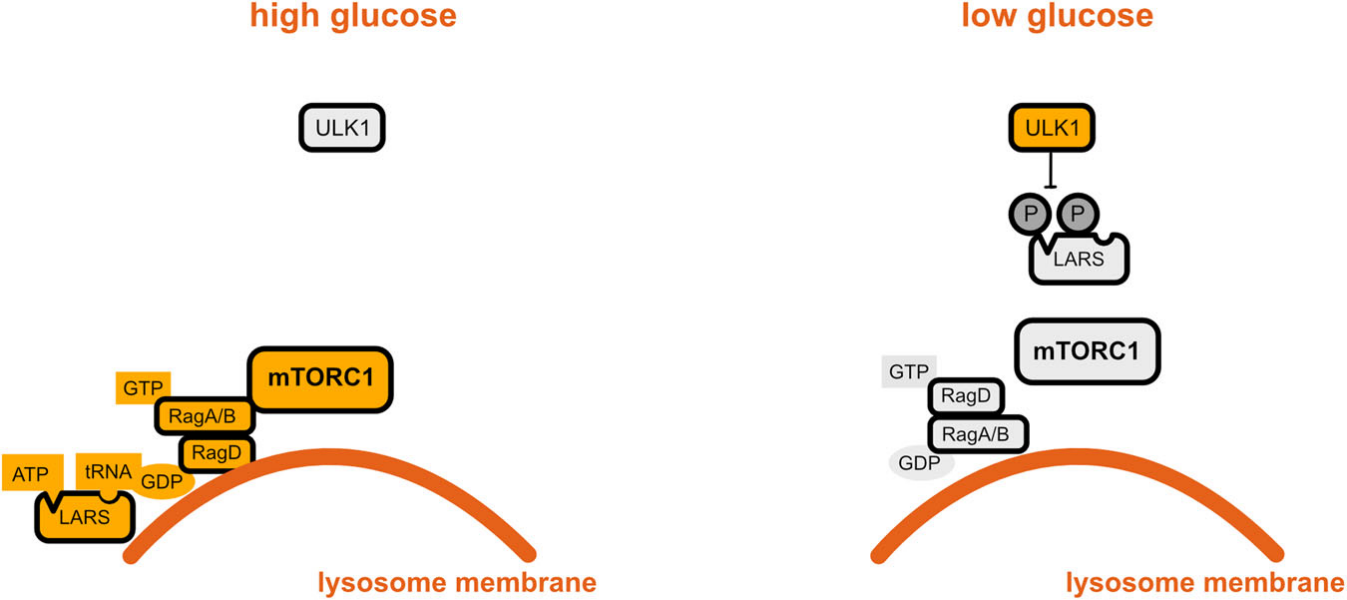
ULK1 and LARS regulate mTORC1 in a glucose-dependent manner. Orange corresponds to active molecules; gray corresponds to inactive molecules.

Leucyl-transfer RNA (tRNA) synthetase 1 (LARS1) is an amino acid sensor signaling leucine levels to mTORC1 by binding to RagD proteins and regulating their GTPase activity^25^. Yoon et al. found that in the absence of glucose, the unc-51 like autophagy activating kinase 1 (ULK1) phosphorylates LARS1 to reduce its binding to leucine, induce its detachment from the lysosomal membranes, and reduce its interaction with RagD, together leading to the inhibition of mTORC1 (Fig. 2), and promotion of leucine catabolism toward ATP generation^26^. Intriguingly, glucose deprivation-induced ULK1 phosphorylation is in part AMPK dependent and previous studies showed that ULK1 is a target of both AMPK and mTORC1 (refs. ^2,27^). Nevertheless, it is still not clear how ULK1 senses glucose starvation^28^.

## ER stress-induced GADD34 as a possible glucose level sensing mechanism

Another protein regulating mTORC1, through TSC1/2, in response to glucose starvation is growth arrest and DNA damage protein 34 (GADD34)^29^. GADD34 is induced during endoplasmic reticulum (ER) stress and functions to downregulate the ER stress response, allowing cells to recover from stress and resume protein synthesis^30^. Notably, upon glucose restriction, GADD34 was reported to be induced by ATF4, and to bind and dephosphorylate TSC2, leading to mTORC1 inhibition^29^ (Fig. 1). Because TSC1/2 inhibition is important for the proper ER stress response^31^ and for survival upon glucose starvation^18^, and because ER stress is induced by glucose starvation^32,33^, it is possible that the induction of the ER stress response (likely through GADD34) is a mechanism by which mTORC1 senses glucose starvation. Nevertheless, *Tsc1*^−/−^ cells failed to induce ATF4 and GADD34 expression, upon induction of ER stress^31^ or glucose starvation^29^ respectively, suggesting that there might be a feedback loop and that mTOR inhibition may be upstream to GADD34 expression.

## The TSC complex component TBC1D7

TBC1D7 was identified as an additional component of the TSC complex whose depletion activates mTORC1 (ref. ^34^; Fig. 1). In addition, while TBC1D7 contributes to mTORC1 inhibition upon glucose starvation by repressing Rheb, this is not the case upon amino acid withdrawal^34^. How TBC1D7 senses glucose starvation is not known.

## Sensing through glycolytic flux

**Fig. 3:**
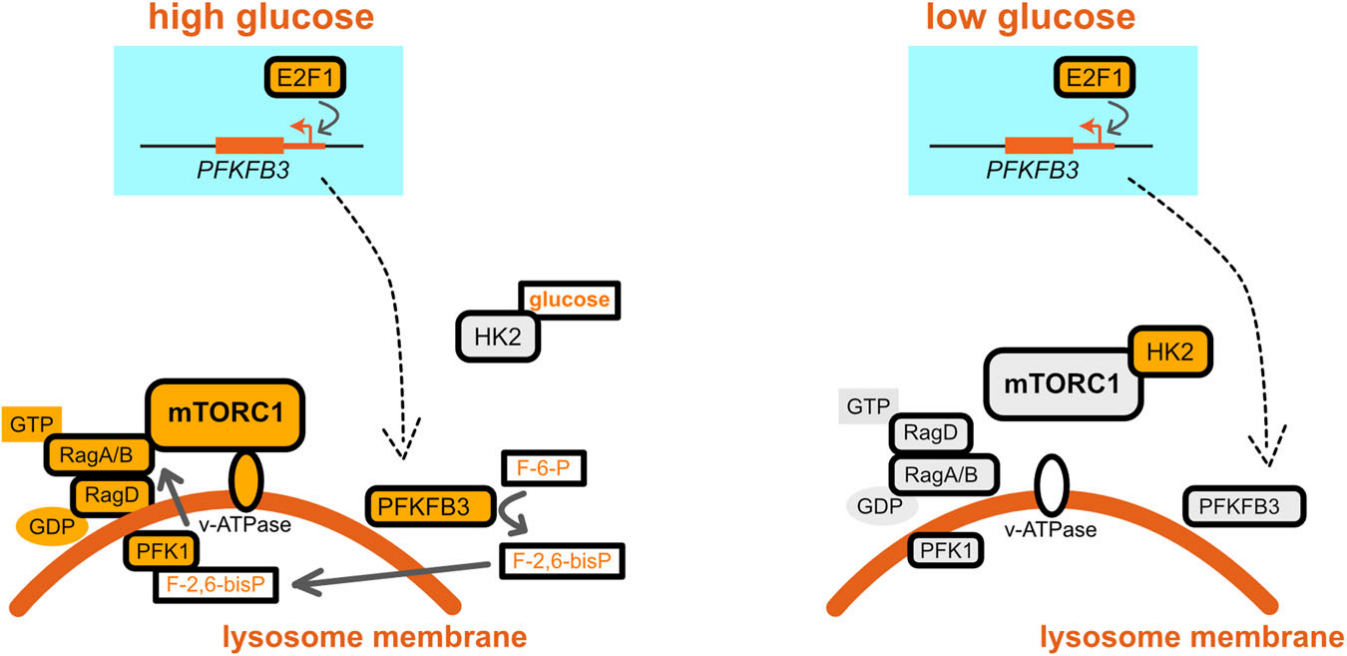
PFKFB3 and HK2 pathways control mTORC1 in response to glucose concentrations. F-6-P is fructose 6-P; F-2,6-bisP is fructose 2,6-P_2_. Orange corresponds to active molecules; gray corresponds to inactive molecules.

E2F1 is a pro-oncogenic transcription factor promoting mTORC1 activity through two distinct mechanisms. On one hand, it was described that E2F1 induces the expression of a v-ATPase subunit to stimulate mTORC1 activity^35^. On the other hand, Almacellas et. al. found that E2F1 induces mTORC1 activity in response to glucose stimulation by promoting the transcription of 6-phosphofructo-2-kinase/fructose-2,6-biphosphatase 3 (PFKFB3), a glycolytic enzyme whose product, fructose 2,6-P_2_, is an allosteric modulator of phosphofructokinase 1 (PFK1), a rate-limiting glycolysis enzyme^36^ (Fig. 3). Interestingly, PFKFB3 activity promotes mTORC1 translocation to the lysosomes by enhancing mTORC1 interaction with RagB. The localization of PFK1, a glycolysis sensor, to lysosomal membranes and the evidence that mTORC1 is sensitive to PFKFB3 activity suggest a mechanism by which mTORC1 may sense glycolytic flux and therefore glucose levels (Fig. 3). Importantly, the same study found that neither E2F1 induction nor PFKFB3 activity affect AMPK activity, excluding AMPK involvement in mTORC1 induction by E2F1 and PFKFB3 (ref. ^36^).

## Hexokinase 2 as a glucose sensor

Upon entering the cytoplasm, glucose binds to and is phosphorylated by hexokinase 1/2 (HK1/2). Roberts et al. found that during glucose starvation, HK2 binds to mTORC1 to inhibit its downstream signaling and induce autophagy^37^ (Fig. 3). In addition, the same authors reported that 2-deoxy-glucose, a known HK and glycolysis inhibitor, blocks HK2 interaction with TORC1, inhibits autophagy and cell survival upon glucose starvation. It is therefore possible that HK2, through an unknown mechanism, acts as a glucose sensor candidate signaling to mTORC1.

## Conclusion

The activity of mTORC1 is tightly regulated by glucose availability through the action of key sensors and signaling components, involving a diverse range of players beyond the prominent energy sensor AMPK. Notably, such regulators only marginally overlap with the ones involved in amino acids-mediated mTORC1 control, highlighting distinct modes of mTORC1 regulation according to the type of nutrients. In a pathological context, in particular cancer, such glucose-responding mTORC1 upstream regulators could represent potential therapeutic targets. Indeed, since the inhibition of mTORC1 is a critical event supporting tumor cell survival under the glucose-deprived conditions commonly encountered in the tumor microenvironment, preventing this blocking by targeting one of its regulators is expected to lead to cell death. Among all the regulators described above, AMPK holds promises as a target as it was shown to promote tumor survival under glucose deprivation^38^ and to support progression of few cancer types, such as glioblastoma, non-small-cell lung carcinoma, and colorectal cancer^39–41^.

## References

[1] Guertin DA, Sabatini DM (2007). Defining the role of mTOR in cancer. Cancer Cell.

[2] Liu, G. Y., Sabatini, D. M. mTOR at the nexus of nutrition, growth, ageing and disease. *Nat. Rev. Mol. Cell Biol.* **21**, 183–203 (2020). 10.1038/s41580-019-0199-y PMC7102936 31937935

[3] Musa J (2016). Eukaryotic initiation factor 4E-binding protein 1 (4E-BP1): a master regulator of mRNA translation involved in tumorigenesis. Oncogene.

[4] Dowling RRJO (2010). mTORC1-mediated cell proliferation, but not cell growth, controlled by the 4E-BPs. Science.

[5] Buttgereit F, Brand MD (1995). A hierarchy of ATP-consuming processes in mammalian cells. Biochem. J..

[6] Leprivier G (2014). Stress-mediated translational control in cancer cells. Biochim. Biophys. Acta.

[7] Choo AY (2010). Glucose addiction of TSC null cells is caused by failed mTORC1-dependent balancing of metabolic demand with supply. Mol. Cell.

[8] Lee C-H (2007). Constitutive mTOR activation in TSC mutants sensitizes cells to energy starvation and genomic damage via p53. EMBO J..

[9] Zhang CS, Hardie DG, Lin SC (2020). Glucose starvation blocks translation at multiple levels. Cell Metab..

[10] Kim J, Guan K-L (2019). mTOR as a central hub of nutrient signalling and cell growth. Nat. Cell Biol..

[11] Nagy JA, Chang SH, Dvorak AM, Dvorak HF (2009). Why are tumour blood vessels abnormal and why is it important to know?. Br. J. Cancer.

[12] Faes S, Demartines N, Dormond O (2017). Resistance to mTORC1 inhibitors in cancer therapy: from kinase mutations to intratumoral heterogeneity of kinase activity. Oxid. Med. Cell Longev..

[13] Teng QX (2019). Revisiting mTOR inhibitors as anticancer agents. Drug Discov. Today.

[14] Palm W (2015). The utilization of extracellular proteins as nutrients is suppressed by mTORC1. Cell.

[15] Hardie DG, Ross FA, Hawley SA (2012). AMPK: a nutrient and energy sensor that maintains energy homeostasis. Nat. Rev. Mol. Cell Biol..

[16] Liu Y (2020). TLR9 and beclin 1 crosstalk regulates muscle AMPK activation in exercise. Nature.

[17] Palomo-Guerrero M (2019). Sensing of nutrients by CPT1C regulates late endosome/lysosome anterograde transport and axon growth. Elife.

[18] Inoki K, Zhu T, Guan K-L (2003). TSC2 mediates cellular energy response to control cell growth and survival. Cell.

[19] Gwinn DM (2008). AMPK phosphorylation of raptor mediates a metabolic checkpoint. Mol. Cell.

[20] Efeyan A (2013). Regulation of mTORC1 by the Rag GTPases is necessary for neonatal autophagy and survival. Nature.

[21] Kalender A (2010). Metformin, independent of AMPK, inhibits mTORC1 in a rag GTPase-dependent manner. Cell Metab..

[22] Sancak Y (2008). The Rag GTPases bind raptor and mediate amino acid signaling to mTORC1. Science.

[23] Huang J, Manning BD (2008). The TSC1-TSC2 complex: a molecular switchboard controlling cell growth. Biochem. J..

[24] Zhang CS (2014). The lysosomal v-ATPase-ragulator complex is a common activator for AMPK and mTORC1, acting as a switch between catabolism and anabolism. Cell Metab..

[25] Han JM (2012). Leucyl-tRNA synthetase is an intracellular leucine sensor for the mTORC1-signaling pathway. Cell.

[26] Yoon I (2020). Glucose-dependent control of leucine metabolism by leucyl-tRNA synthetase 1. Science.

[27] Egan DF (2011). Phosphorylation of ULK1 (hATG1) by AMP-activated protein kinase connects energy sensing to mitophagy. Science.

[28] Lehman E, Abraham RT (2020). A sugary input to leucine sensing. Science.

[29] Watanabe R (2007). GADD34 inhibits mammalian target of rapamycin signaling via tuberous sclerosis complex and controls cell survival under bioenergetic stress. Int. J. Mol. Med..

[30] Novoa I, Zeng H, Harding HP, Ron D (2001). Feedback inhibition of the unfolded protein response by GADD34-mediated dephosphorylation of eIF2α. J. Cell Biol..

[31] Kang YJ, Lu MK, Guan KL (2011). The TSC1 and TSC2 tumor suppressors are required for proper ER stress response and protect cells from ER stress-induced apoptosis. Cell Death Differ..

[32] Xi H, Barredo JC, Merchan JR, Lampidis TJ (2013). Endoplasmic reticulum stress induced by 2-deoxyglucose but not glucose starvation activates AMPK through CaMKKβ leading to autophagy. Biochem. Pharm..

[33] Ye J (2010). The GCN2-ATF4 pathway is critical for tumour cell survival and proliferation in response to nutrient deprivation. EMBO J..

[34] Dibble CC, Cantley LC (2015). Regulation of mTORC1 by PI3K signaling. Trends Cell Biol..

[35] Meo-Evoli N (2015). V-ATPase: a master effector of E2F1-mediated lysosomal trafficking, mTORC1 activation and autophagy. Oncotarget.

[36] Almacellas E (2019). Phosphofructokinases axis controls glucose-dependent mTORC1 activation driven by E2F1. iScience.

[37] Durán RV (2012). Glutaminolysis activates Rag-mTORC1 Signaling. Mol. Cell.

[38] Liang J, Mills GB (2013). AMPK: a contextual oncogene or tumor suppressor?. Cancer Res.

[39] Chhipa RR (2018). AMP kinase promotes glioblastoma bioenergetics and tumour growth. Nat. Cell Biol..

[40] Eichner LJ (2019). Genetic analysis reveals AMPK is required to support tumor growth in murine kras-dependent lung cancer models. Cell Metab..

[41] Wang Y-N (2020). AMPKα1 confers survival advantage of colorectal cancer cells under metabolic stress by promoting redox balance through the regulation of glutathione reductase phosphorylation. Oncogene.

